# Monthly Dynamics of Plasma Elements, Hematology, Oxidative Stress Markers, and Hormonal Concentrations in Growing Male Shiba Goats (*Capra hircus*) Reared in Tokyo-Japan

**DOI:** 10.3390/ani12050645

**Published:** 2022-03-03

**Authors:** Ahmed S. Mandour, Haney Samir, Marwa A. El-Beltagy, Lina Hamabe, Hend A. Abdelmageed, Izumi Watanabe, Ahmed Elfadadny, Kazumi Shimada, Gamal El-Masry, Salim Al-Rejaie, Ryou Tanaka, Gen Watanabe

**Affiliations:** 1 Department of Animal Medicine (Internal Medicine), Faculty of Veterinary Medicine, Suez Canal University, Ismailia 41522, Egypt; 2 Laboratory of Veterinary Surgery, Tokyo University of Agriculture and Technology, Tokyo 183-0054, Japan; linahamabe@vet.ne.jp (L.H.); kazumi-s@go.tuat.ac.jp (K.S.); ryo@vet.ne.jp (R.T.); 3 Department of Theriogenology, Faculty of Veterinary Medicine, Cairo University, Giza 12211, Egypt; haneyvet360@cu.edu.eg; 4 Laboratory of Veterinary Physiology, Tokyo University of Agriculture and Technology, Tokyo 183-0054, Japan; gen@cc.tuat.ac.jp; 5 Department of Biochemistry, Faculty of Veterinary Medicine, Suez Canal University, Ismailia 41522, Egypt; marwa_beltagui@vet.suez.edu.eg; 6 Department of Bacteriology, Animal Health Research Institute, Agriculture Research Center, Ismailia Laboratory, First District, Ismailia 41511, Egypt; hendabdelmageed312@gmail.com; 7 Cooperative Department of Veterinary Medicine, Faculty of Agriculture, Tokyo University of Agriculture and Technology, Tokyo 183-8509, Japan; 8 Laboratory of Environmental Toxicology, Tokyo University of Agriculture and Technology, Tokyo 183-8509, Japan; wataizumi@cc.tuat.ac.jp; 9 Department of Animal Internal Medicine, Faculty of Veterinary Medicine, Damanhour University, Damanhour 22511, Egypt; ahmed.elfadadny@vetmed.dmu.edu.eg; 10 Agricultural Engineering Department, Faculty of Agriculture, Suez Canal University, Ismailia 21522, Egypt; gamal.elmasry@agr.suez.edu.eg; 11 Department of Pharmacology & Toxicology, College of Pharmacy, King Saud University, Riyadh 11564, Saudi Arabia; rejaie@ksu.edu.sa

**Keywords:** antioxidants, plasma elements, reproductive hormones, Shiba goats, hematobiochemical, puberty

## Abstract

**Simple Summary:**

During the first stage of an animal’s life, various physiological alterations with the concomitant development of different body organs occur. This period is also accompanied by different kinds of stressors, including, but not limited to, the stress of weaning, metabolic change, and peri-puberty changes in reproductive functions. Shiba goats, the main goat breed in Japan, are not commonly used as food animals. However, male Shiba goats largely contribute to reproductive and cardiology research activities, as well as being used for educational purposes for elementary school children. The physiological data regarding laboratory measurements in Shiba goats are lacking, especially at a young age. In this study, we investigated the age-related changes in hematology, plasma mineral concentrations, hormones, and oxidative stress markers during the first five months (neonatal and peri-puberty age) in male Shiba goats.

**Abstract:**

From a clinical point of view, the establishment of laboratory variables during the first few months of an animal’s life helps clinicians to make sure they base their medical decisions on laboratory values for the specific breed and age group. The present study aimed to investigate the monthly dynamics in some plasma elements, hematology, reproductive hormones, and oxidative stress marker profiles during the first five months of age (neonatal and peri-puberty stage) in male Shiba goat’s kids. Sixteen kids were investigated from the first to the fifth month (M1 to M5), and the data were presented as the statistical difference between them. Whole blood and plasma samples were collected monthly for analysis of basal hematology, plasma elements concentration (trace elements: Cu, Zn, Se, Fe, and Cr; macroelements: Ca and Mg), circulating hormones (cortisol, FSH, LH, IGF1, immunoreactive inhibin, testosterone, T3, and T4), and oxidative stress markers (MDA, CAT, SOD, and GPX). The results showed age-related changes in the observed parameters. The fifth month recorded the lowest level of almost all investigated minerals, except for Cr. Plasma hormone levels revealed age-dependent increases in IGF-1 and testosterone, age-related decreases in T3 and T4, and non-significant changes in cortisol and FSH. Besides, the concentrations of inhibin and LH were significantly higher at M1–M3 compared with M4–M5. Plasma SOD, GPX, and CAT were increased with age. In conclusion, age-related changes and a distinction of age in months was found necessary to interpret the laboratory results, specifically in terms of age in months and the peri-puberty stage in young goats, which are important to follow up the age-specific diseases, reproductive status, and treatment follow-ups in this stage.

## 1. Introduction

The goat is one of the oldest domesticated animals and considered the greatest living species adaptable to the hot and dry regions of the world. Japanese Shiba goats are an efficient animal model in studying the pathophysiology of reproduction, cardiovascular, and hemodynamics of ruminants [1,2,3,4]. Rather than being food animals, Shiba goats possess another great importance in Japan, since they are temporarily kept in elementary schools for children’s entertainment, in addition to the educational and psychological improvement of young generations [5]. 

The health status and performance of an animal are affected by nutrient availability. It has been demonstrated that essential elements that are required for proper functions and homeostasis can have equally beneficial or detrimental effects depending on their balance [6]. They act synergistically at the proper concentration in the protection of the host homeostasis and prevailing defense mechanisms, as well as reproduction processes from gametogenesis to puberty [7,8,9]. There is a complexity in the mode of action by which these elements affect animal health and performances, including metallobiomolecules and the neuro-hormonal relationship [6,10], which are indispensable for the normal function of the immune system, hematopoietic tissue, skin and coat, ovarian folliculogenesis, spermiogenesis, embryo development, and increased pregnancy rates [11,12,13]. There is a wide range of clinical abnormalities related to mineral deficiency in goats, which are collectively associated with fetal losses, poor productive and reproductive performances, locomotor disturbances, and deteriorative changes in skin and coat [14]. 

Assessment of mineral elements and their roles in oxidative stress are essential as a complementary tool in the evaluation of the nutritional and metabolic status of animals. Several studies have demonstrated the link between nutritional status, oxidative stress markers, and changes in organ and tissue contents of some mineral elements at normal and deficient levels concerning puberty onset, impaired reproductive efficiency, reproductive seasonality, growth rate, milk production, and mortality of goats and their kids [12,15,16,17]. The first few months of an animal’s life are critical because they determine the performance style and the economic trait of the individuals; hence, more benefits would be obtained when earlier puberty and sexual maturity, which are ultimately under the control of reproductive hormones. Abdolvahabi et al. [18] suggested that specific age-related reference values are essential for goats for the proper interpretation of laboratory results. 

Dynamics in hematobiochemical parameters greatly contribute to the performance and health status of small ruminants during the early postnatal and the rapidly growing periods of life [19]. Copious reports have investigated the hematological and biochemical variables of calves [15,20,21,22,23], lambs [24], and goat’s kids [25] in comparison with adult animals in the same species or even between species; however, monitoring the longitudinal changes throughout age during the growing period in goats is still limited [26], and more investigations are required. In addition, reference intervals for laboratory variables are commonly established for adult animals that may be misleading if used to evaluate values from young individuals. The present study aims to investigate the age-related changes in the hematological profile, plasma elements concentration, reproductive hormones, and oxidative stress markers during the first five months of age to uncover the laboratory changes in these parameters during the neonatal and peri-puberty period in male Shiba goat’s kids. 

## 2. Materials and Methods

### 2.1. Animals 

A total of 16 newborn miniature male Shiba goat’s kids obtained from 10 dams (6 dams gave twins, 4 dams yielded a single kid) were included in this study. All mothers became pregnant after natural mating without estrous synchronization and recorded no history of disease during pregnancy, abortion, dystocia, or post-parturient diseases. All kids were born in winter (February), and the investigation ended by June 2017. Kids were kept with their mothers under natural photoperiod and temperature in an adequately constructed goat barn belonging to the Laboratory of Veterinary Physiology, Tokyo University of Agriculture, Fuchu campus, Japan. The latitude and longitude of Fuchu city, where the study was conducted, are 35.67° N and 139.48° E, respectively (travelmath.com, accessed on 4 November 2021). Local climatic conditions, including average environmental temperature, relative humidity (RH%), wind-speed, and temperature-humidity index (THI), concerning the sampling times, which correspond to the age of kids in months, were retrieved from the local Japan Meteorological Agency (Tokyo, WMO station, ID: 47,662) and are presented in Table 1. 

Special kid’s cages were provided to mothers and kids during the first month. Kids were allowed their mother’s milk *ad libitum* until the age of 8 weeks and were separated from mothers at the end of the second month. From the start of the second month, 0.3 kg of crushed alfalfa hay cubes were provided to the kids, and the quantity was increased after separation from dams according to the requirement for growth (0.4 kg/twice daily). Licks of mineralized salt and water were provided *ad libitum*. Chemical analysis of the diet was done using standard methods and is illustrated in Table 2. Male Shiba goats show nonseasonal breeding activities under the natural photoperiod conditions in Tokyo prefecture, Japan, where the current study was performed. The puberty age in male Shiba goats occurs from 3 to 4 months of age [27]. The study began from the first month of life and was terminated at the end of 5 months in all kids to cover the neonatal and peri-puberty stages. 

### 2.2. Clinical Examination

The health status of animals was monitored throughout the study period, which lasted for five successive months, and the clinical data were recorded based on the clinical examination protocol of goats [28]. Neither case history nor clinical examination revealed any clinical abnormality in the investigated group throughout the study period. The physiological parameters of newborn kids are illustrated in Table 3. 

### 2.3. Blood Samples and Hematology

Jugular venous blood samples were collected at the end of each month for five consecutive months. Two mL of whole blood in an ethylenediaminetetraacetic acid (K_3_EDTA) was used for hematological analysis using the automatic cell counter (Celltac Alpha MEK-6400, Nihon Kohden, Tokyo, Japan). To obtain plasma samples, five mL of whole blood in heparinized tubes (Venoject II, Terumo, Tokyo, Japan) was collected and centrifuged at 3000 rpm for 15 min, and the clean non-hemolyzed plasma was collected and kept at −20 °C for analysis of elements, hormones, and oxidative stress markers. The reported hematological parameters include the following measurements: hemoglobin concentration (Hb), red blood cell count (RBC), hematocrit (HCT), mean corpuscular volume (MCV), mean corpuscular hemoglobin (MCH), mean corpuscular hemoglobin concentration (MCHC), total white blood cell count (WBC), red blood cell distribution width (RDW), platelet count (PLT), plateletcrit (PCT), mean platelet volume (MPV), and platelet distribution width (PDW). Modified Wright’s stain was used to perform the differential leukocytic count using a digital leukocytic calculator [29].

### 2.4. Analysis of Plasma Elements Concentration

#### 2.4.1. Preparations of Samples, Blank and Standard 

Plasma concentrations of trace elements (Cu, Zn, Se, Fe, Cr, Mo, and Co) and macroelements (Ca, Mg) were determined using the previously described dry ashing protocol [3]. In brief, an aliquot of plasma was put into vials and allowed to dry overnight in a hot air oven at 100 °C. Dried samples were chemically treated with concentrated nitric acid (2 mL), followed by microwave digestion (200 °C for 10 min). The blank (nitric acid) was prepared with the same procedures applied to the plasma samples. Samples and blank were filtered and diluted in polyethylene tubes using milli-Q water. Serial dilution (0.0, 0.005, 0.01, 0.05, 0.1, 0.5, 1.0, 5 ppm) of the multi-element standard (Agilent, Hachioji, Japan) was carried out. Element analysis was performed by inductively coupled plasma mass spectrometry (ICP-MS; Agilent 7500, Agilent, Hachioji, Japan) with a manual sampler. ICP-MS setting conditions and measured isotopes are described in Table 4.

#### 2.4.2. ICP-MS Quality Control and Data Calculations

A quality assurance procedure was performed following the previously described protocols [30,31,32]. The accuracy of the analytical procedures was verified using standard reference material (Bovine liver, NIST-RM 1577b, Gaithersburg, MD, USA), which was used to estimate the reproducibility of various elements. The relative standard deviations (RSD) of the analyzed elements examined by sextuplicate analyses of NIST 1577b were less than 10%, and the recovery rate of selected elements was ranged between 90.2 and 105%. A multi-element standard solution as an external standard (Agilent, Hachioji, Japan), rhodium as an internal standard (Wako Pure Chemical Industries, Osaka, Japan), and a tuning solution (Agilent Technologies, 5301 Stevens Creek Blvd, Santa Clara, CA, USA 95051) were used for ICP-MS calibration. All samples were corrected against the blank and were analyzed in triplicate. A freshly prepared working calibration standard solution was prepared by appropriate dilution of the multi-elemental standards with nitric acid. Element quantification was determined using ICP-MS-specific Work Station Software (MassHunter, version A.8.01.01 Agilent Technologies, Inc. 2012, Tokyo, Japan).

The difference between wet weight and dry weight, as well as the water content of samples, was calculated. Mathematical calculation of the standard curve, limits of detection (calculated as 3 times the standard deviation of the blanks), and concentration of elements in PPM were performed using an excel worksheet. Serial dilution of the multi-element standard was used in the calculation of the standard curve of each element through the Trend function in the Microsoft Excel worksheet.

To compare our results with element levels from previous studies, the obtained data (determined as PPM on a dry weight basis) were converted into wet weight basis (mg/L) using the water content values through the following equations [31]:

(1) Water content = wet weight − dry weight/(wet weight ×100).

(2) The dry weight of each element (PPM)= dilution factor × standard curve

(3) The wet weight of each element (mg/L)=((dry weight ×(100− water %)/100)

### 2.5. Plasma Hormonal Assays

The concentration of plasma hormones was determined in triplicate using a double-antibody radioimmunoassay system using I125-labeled radioligands. All procedures were done in the same laboratory at Tokyo University of Agriculture and Technology, Japan. Concentrations of plasma cortisol were determined using rabbit anti-cortisol (HAC-AA71-02RBP85), cortisol-3-CMOBSA (80-IC20) for iodination, and the reference standard of hydrocortisone (H-4001). Plasma concentrations of testosterone, cortisol, follicle-stimulating hormone (FSH), luteinizing hormone (LH), and immunoreactive inhibin (INH) were measured as described by previous studies [33,34,35,36]. Concentrations of testosterone (ng/mL) were measured in plasma using specific antisera (GDN 250). Plasma concentrations of FSH (ng/mL) were measured using anti-ovine FSH, NIDDK-oFSH-RP-1 as a standard reference, and NIDDK-FSH-I-1 for radio-iodination, while plasma LH (ng/mL) was measured using anti-ovine LH (YM 18), NIDDK-oLH-RP-24 as a reference standard, and NIDDK-oLH- I-3 for radio-iodination. Plasma concentration of inhibin (ng/mL) was measured using anti-bovine antiserum (TNDH-1) and bovine 32-kDa inhibin for radio-iodination. The intra- and inter-assay coefficients of variation were 9.4 and 2.4% for cortisol, 8.2 and 9.2% for T, 9.6 and 11.8% for oFSH, 5.6 and 6.8% for oLH, and 4.2% and 12.3% for INH, respectively. Insulin-like growth factor 1 concentration (IGF-1; ng/mL) was measured using ethanol acid cryo-precipitation method according to the protocol of Mizukami et al. [37] and the modified steps by [38]. Anti-serum against human IGF-1 (code AFP4892898; raised in rabbits) and recombinant human IGF-1 (as a reference standard and for radioiodination) were used. The intra- and inter-assay coefficients of variation were 8.7 and 5.1%, respectively. Goat triiodothyronine (T3) and tetraiodothyronine (T4) were measured by ELIZA kits (MyBioSource, San Diego, CA, USA, Cat No. MBS265631, MBS703630). 

### 2.6. Assessment of Oxidative Stress Markers 

Plasma activity of malondialdehyde (MDA), catalase (CAT), superoxide dismutase (SOD), and glutathione peroxidase (GPX) were measured using a commercial colorimetric test (Biodiagnostic Co, Giza, Egypt). MDA was measured according to the modified method of Nielsen et al. [39]. MDA formed during the decomposition of lipid peroxidation products was measured by the reaction of thiobarbituric acid reactive substances (TBARS) with thiobarbituric acid (TBA). CAT activity was measured through the continuous spectrophotometric degradation of H_2_O_2_ at 520 nm using a redox dye [40]. The GPX activity in plasma was measured according to the method of Paglia and Valentine [41], which is based on the oxidation of reduced glutathione (GSH) to oxidized glutathione (GSSG) catalyzed by GPX. GPX activity was measured based on NADPH consumption in the enzyme-coupled reactions through the standard equation. SOD activity was assayed using the method of McCord and Fridovich [42], after modifications. SOD scavenges the superoxides provided by xanthine oxidase. All measurements were done according to manufacturer protocols provided in the kits. Analysis was done using a semi-automated analyzer photometer 5010 V5+ (Robert Riele GmbH, Berlin, Germany). The quality control of each parameter was determined based on the calculation of the standard curve. The optical density (OD) of the provided standard and blank specific for each assay were determined. The slope of the standard curve was created by blotting the standard concentrations against the ∆OD in each assay (R^2^ > 0.99), and the color intensity at the specified wavelength was directly proportional to the concentration in the sample. The ∆OD was used to determine the activity of the target in the sample from the generated standard curve.

### 2.7. Statistical Analysis

All data were categorized depending on the age of kids in months (M1, M2, M3, M4, M5) and used for statistical analysis. The sample size was measured based on the outcomes and calculation performed with G*Power 3.1.9.2 software [43], assuming a moderate effect of age on the reported measurements with a 0.30 effect size [44]. The normality test of all measurements was carried out using the Kolmogorov–Smirnov (KS) test. All measurements were analyzed using repeated one-way analysis of variance using Friedman’s test. Bonferroni’s multiple comparison test was used as a post-hoc to determine the age-related changes in all parameters (*p* < 0.05). Spearman’s correlation analysis between reported parameters was performed. All analyses were carried out using GraphPad Prism 8.0 (GraphPad Software, San Diego, CA, USA). All data were graphed and presented as the median and interquartile range. The numerical values of reported data are presented in Supplementary Table S1. 

## 3. Results

### 3.1. Age-Related Changes in Hematology 

Table 5 illustrates the hematological profile. There were no significant changes in RBC, Hb, MCV, MCH, MCHC, and WBC throughout the investigated period (*p* > 0.05). The HCT concentration at M1–M3 was higher than M4 (*p* = 0.013). RDW at M1–M2 was higher than M4–M5 (*p* = 0.007). Moreover, the highest PLT count was observed at M3–M4 (*p* = 0.018); meanwhile, the greatest MPV and PDW were observed at M1. Concerning the differential leukocytic count, a significantly higher neutrophil concentration at M4 compared with M2, and a significant increase in the lymphocytic count at M2 and M3 compared with M1, was observed. Increased monocyte and eosinophil count at M1 compared with the preceding age groups was also observed (*p* = 0.005, *p* = 0.001, respectively). 

### 3.2. Age-Related Changes in Plasma Elements Concentration 

**Figure 1:**
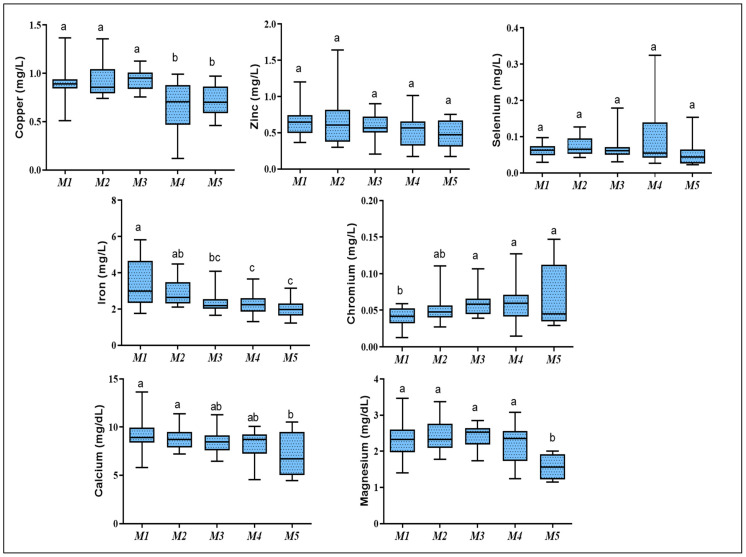
Whisker box plots showing the median (central horizontal line) and interquartile range (25% downward, 75% upward bars) of plasma element concentrations in male Shiba goat’s kids during the first five months of life. The upper and lower ends of the whiskers show the maximum and minimum values. Different letters indicate a significant difference between ages per month (*p* < 0.05).

Data for element concentrations are presented in Figure 1. Plasma concentrations of Co, Mn, and Mo were lower than the limit of detection of the IC-PMS, and their values were excluded. Plasma Cu concentration showed a significant reduction at M4–M5 compared with M1–M3 (*p* < 0.0001). Plasma Fe concentration showed a regular pattern of reduction with the advance of kid’s age and its lowest level was observed at M5, which was significantly lower than M1 (*p* = 0.0001). In contrast, Cr revealed a regular pattern of increment with age advance, and a significant increase in Cr level from M3 to M5 compared with M1 was noticed (*p* < 0.05). The level of Se and Zn showed no significant change with age. Plasma Ca and Mg levels were significantly decreased at M5 compared with earlier months (*p* = 0.006 and *p* = 0.000, respectively).

### 3.3. Age-Related Changes in the Concentration of Plasma Hormones

**Figure 2:**
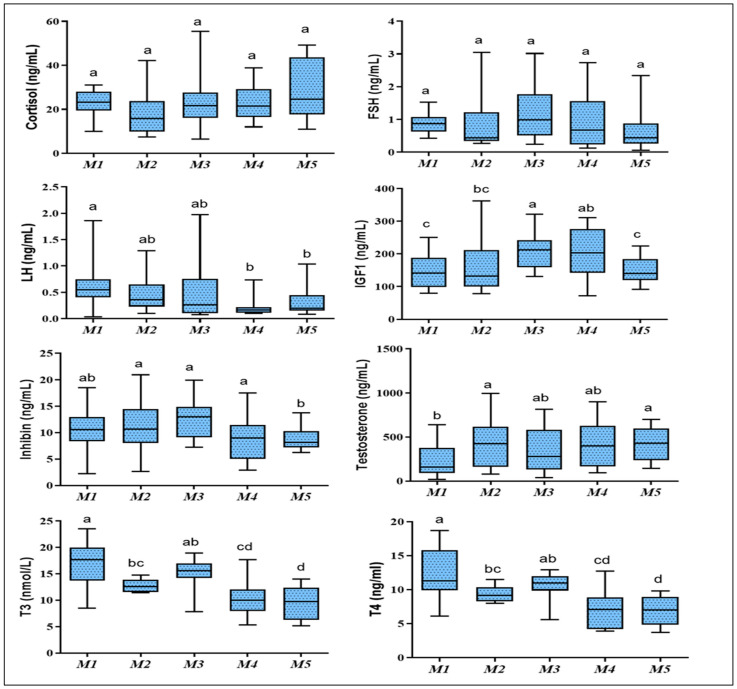
Whisker box plots showing the median and range of plasma hormone concentrations in newborn goat’s kids during the first five months of life. Different letters indicate a significant difference between ages per month (*p* < 0.05). FSH, follicle-stimulating hormone; LH, luteinizing hormone; IGF1, insulin-like growth factor 1; T3, triiodothyronine; T4, tetraiodothyronine.

Figure 2 summarizes the hormonal changes. The concentration of cortisol and FSH showed non-significant change among the different studied ages. There were age-dependent increases in the concentration of IGF-1, and the maximum concentrations were observed at M3 compared with other months. The maximum concentration of testosterone was observed at M5, followed by M2, which was significantly higher than M1 (*p* = 0.045); however, there were no significant changes from M2 to M5. Concentrations of inhibin and LH were significantly higher at pre-puberty age (M1–M3) compared with M4–M5. In the current study, the concentrations of inhibin in the plasma during the first 5 months of age were also correlated positively with the levels of FSH and negatively with the levels of testosterone. Highly significant decreases in an age-dependent manner were observed in the concentrations of T3 and T4 (*p* = 0.0001, both).

### 3.4. Age-Related Changes in the Level of Oxidative Stress Markers 

**Figure 3:**
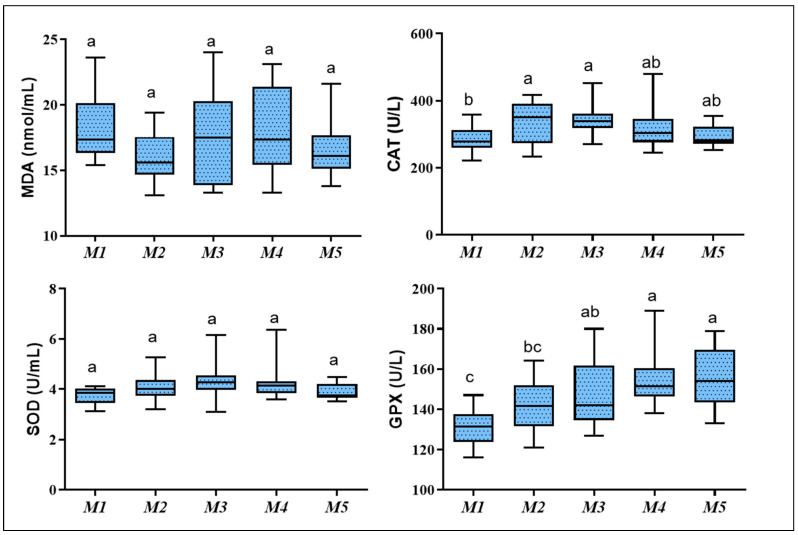
Whisker box plots showing the median and range of plasma level of oxidative stress markers in male Shiba goat’s kids during the first five months of life. Different letters indicate a significant difference between ages per month (*p* < 0.05). CAT, catalase; MDA, malondialdehyde; SOD, superoxide dismutase; GPX, glutathione peroxidase.

Figure 3 summarizes the plasma concentration of oxidative stress markers. The GPX showed a regular trend of increasing with age advance, whereas the level of GPX at M4–M5 was significantly higher than M1 to M2 (*p* = 0.001). In contrast, the highest concentration of plasma CAT was observed at M2 and M3, which was higher than M1. SOD and MDA showed no significant differences (*p* > 0.05). 

### 3.5. Correlation Analysis between the Measured Variables

**Figure 4:**
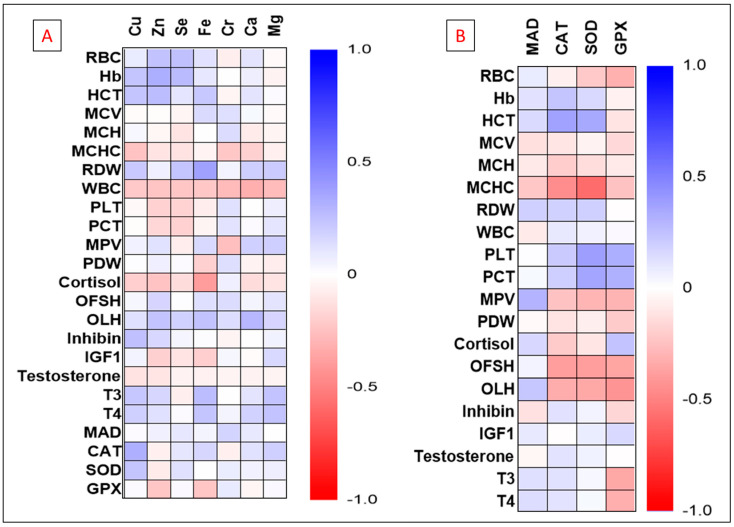
Heatmap illustrating the correlation results between plasma element concentrations (Ca, Mg, Cu, Zn, Se, Cr, Fe) and hematology, oxidative stress markers, and plasma hormonal profile (**A**). The relationship between oxidative stress markers (MAD, SOD, GPX, CAT) and hematology and hormonal profile throughout the study period was also presented (**B**).

Correlation results between plasma elements and obtained measurements, as well as the correlation between oxidative stress markers and other variables, are illustrated in Figure 4. The data revealed a variable correlation between obtained measurements. The level of plasma Ca was positively and significantly correlated with other plasma elements (r > 0.435; *p* < 0.001). Fe was correlated with Cu and Zn (r = 0.345, 0.478; *p* < 0.05). Cr was significantly correlated with Zn, Se, and Mg levels (r = 0.272, 0.513, 0.417; *p* < 0.05). Zn showed a positive correlation with Hb and HCT (r = 0.308, 0.323; *p* < 0.05). 

Plasma Cu concentration was correlated with plasma T3 and T4 levels (r = 0.379, 0.374; *p* < 0.05). There was a negative trend correlation between Se level and T3 and T4. In addition, the level of Ca, Fe, Cu, and Zn showed a negative trend correlation with plasma cortisol (*p* = 0.06). 

Plasma CAT, SOD, and GPX were positively correlated with BW (r = 0.340, 0.314, 0.527; *p* < 0.05) and negatively correlated with FSH (−0.348, −0.305, −0.305; *p* < 0.05) and LH (r = −0.299, −0.213, −0.371; *p* < 0.05), respectively. GPX showed a reverse relationship with T3 and T4 (r = −0.327, −0.295; *p* < 0.05). 

## 4. Discussion

Complete blood cell count, elements profile, antioxidant status, and hormone levels are important in identifying various diseases, reproductive states, and the response to medication. In farm animal practice, a successful management strategy keeps acceptable levels of biologically active components including, but not limited to, balanced minerals that are incorporated into vital physiological pathways, antioxidant defense mechanisms, and hematological and reproductive hormones at functional levels. The synergistic and complementary activity of the aforementioned parameters enhances to some extent the lifelong production and well-being of farm animals [45]. Various studies have profiled the hemato-biochemical variations in different breeds of goats in different geographical areas [16,18] [17]. Nevertheless, Shiba goats—a non-seasonal breeding goat that is reared principally in Japan [1,46], supports lifelong research studies in animal reproduction [1,36], and has been recently introduced in cardiovascular pathophysiology [2,3,4,10]—have not been investigated before. 

In the current study, there were some age-related changes in some hematology, elements, hormonal profile, and oxidative stress markers. The hematological profile in this study revealed non-significant changes in the principal components of basic hematology (RBC, Hb, MCV, MCH, MCHC, and WBC). However, HCT, PDW, PLT, PDW, monocytes, eosinophils, and lymphocytes showed significant differences from the first to the fifth month of life. There is a shortage in age-specific hematobiochemical indices and reference intervals in young goat kids during the investigated stage. Our results are consistent with previous studies [20,21,47], which reported age-associated changes in hematological parameters up to 6 months of age in calves. Moreover, RBCs and MCV coincided with a previous report that showed the same range of RBCs of goats under one year with the proceeding age groups [47]. There are some differences in red cell parameters in our study compared with the previous intervals in adult goats. Hb, PLT, and HCT are consistent; MCV, MCH, and MCHC are lower; and PLT count is higher than values in adult male Shiba goats two years old [3]. In our study, compared with another study, RBCs count was higher and the MCV was lower than adult male goat’s values at 2.5 years old [2]. In addition, MCV and MCH were higher than the previous reference interval in Iraqi goats, but other hematological values were in the same interval [48], indicating that adult male reference values can be used to evaluate Hb, PLT, and HCT in goats up to 5 months, but other parameters are different from adult values.

The MCV and MCHC in goats fluctuated mostly depending upon RBC, Hb, and HCT values. Variation in RBC count could be related to the oxygen-carrying capacity of the blood, which is higher in the first few months of life in goats compared with older ages, and a normal Hb concentration in young goats is maintained by a higher RBC count to compensate for lower MCV [26,29]. Higher HCT in early months was correlated with RBC count. In our study, the decline in HCT at M4 was associated with the lowest RBC count. Kim et al. [23] attributed the reduction in HCT in calves from 16 weeks of age to the inability of animals to produce RBC at a rate equal to the rate of removal from circulation.

The lymphocytic count was significantly increased from the second month and remained higher than at the first month of age, but the WBC remained unchanged. The lymphocytes percentage in yearling goats was higher than the proceeding age [49]. Along with the study intervals, the lymphocytic percentage was higher than the neutrophils, in contrast to the previous study in calves. Changes in leukocytes are connected with suitable protection systems, due to immune variations in animals [23].

Multi-elements analysis is necessary to uncover elements interaction, toxicity, and antagonism. ICP-MS is commonly used for this purpose which provide massive information regarding multiple element concentrations through one-shot analysis of acid-digested plasma samples [50]. Our results revealed a significant reduction in Cu and Fe concentrations in the peri-puberty at age M4 and M5 compared with M1 to M2. This may be attributed to the reduction in the availability of Cu in newborn kids after weaning. Before the development of a functional rumen, Cu absorption is high (70–85%) in milk-fed lambs but decreases to less than 10% after weaning. Changes in the availability of Cu before and after weaning might be related to several factors, including the development of ruminal microflora and subsequent production of sulfide that reacts with Cu to form Cu-sulfide complex, and irreversible adsorption of Cu by fiber particles and microbial cell walls [51,52]. Moreover, there is a close relationship between Cu and Fe homeostasis. Hence, Cu is important for the incorporation of Fe in Hb, intestinal absorption of Fe, and the transfer of Fe from tissues to plasma [53,54]. Therefore, reduced serum Fe concentration with age advance in kids was found to be related to the changes in Cu concentration at this time point [2,55]. The level of plasma Fe was significantly reduced with the advance of a kid’s age; meanwhile, Zn and selenium were apparently decreased. During the early postnatal period, colostrum contains high Zn concentration, which is reduced subsequently when kids changed to normal goat’s milk [56]. Besides, Barrionuevo et al. [57] stated that goat’s milk has an important and beneficial effect on the bioavailability of Cu, Zn, and Se during the early stage of life. Young animals have a greater need for iron and zinc compared to older animals. Zinc level steadily drops in neonatal animals toward adolescence, since growing animals need more zinc than non-growing animals; while the increased demand for iron in earlier months is necessary to replace fetal hemoglobin with normal hemoglobin [58,59].

Plasma Se concentration was almost the same from M1–M3 and showed a slight reduction from M4 to M5. A reduction in Se bioavailability with the advance of age due to the development of ruminal microflora was pointed out [55], which results in chemical reduction of dietary Se to insoluble forms such as elemental Se or selenides [60,61,62]. Generally, Se is mainly present in GPX in red blood cells, and blood Se concentration remains constant before dropping after 6 months of age in calves [63]. Our results showed a gradual age-dependent increase in Cr concentration. There is no sufficient data reviewing either reference ranges of plasma Cr concentration or its availability in goats. The biological functions of the Cr are related to the glucose tolerance factor, which is vital for carbohydrate metabolism, potentiates the action of insulin, and normalizes blood sugar levels [64]. Therefore, the age-related increment of Cr concentration may be attributed to the increased food intake and enhanced metabolism. Overall, variation in plasma trace elements concentration is possibly related to changes in liver elements concentration with age, which is greater in young animals than adults [65]. In our study, Mg and Ca were reduced at M5. Young goats have higher serum Mg and Ca concentrations than adults, as the availability of Mg in ingested food increases Mg utilization for bone mineralization in growing neonates [66]. Besides, Ca is essential for bone formation, and a high concentration of Ca in the growing period is related to the ability of young goats to keep a high level of vitamin D biosynthesis in the skin, which subsequently increases Ca absorption [67], in addition to high parathyroid functions and milk intake in the first few months after birth [22]. Moreover, the level of Ca is relatively high in goats less than 6 months, while Mg is lower in juvenile goats (between 4–6 months) compared with adults [68]. The observed correlation between measured elements in our study was in accordance with previous studies [65]. 

Reproduction in mammals is orchestrated by complex neuroendocrine networks, starting from early postnatal life to the peri-pubertal age and integrating a wide range of internal and external signals for the release of sex hormones under the control of the hypothalamic-pituitary-gonadal axis (HPG) [69]. The HPG system primarily emerges to control the secretion of gonadotropin-releasing hormone (GnRH), gonadotropin hormones, and subsequently gonadal function. The pulsatile secretion of hypothalamic gonadotropin-releasing hormone (GnRH) stimulates the synthesis and release of follicle-stimulating hormone (FSH) and luteinizing hormone (LH) by pituitary gonadotropes [70]. In the current study, there were increases in the concentrations of plasma testosterone at M2 onward compared with M1, and the maximum concentrations were at M5. These results were in agreement with the previous studies of [71,72]. Male Shiba goats reach puberty at about 3–4 months of age [27]. Therefore, increases in testosterone concentrations may be attributed to puberty attainment. Similarly, we found an age-dependent increase in the concentrations of IGF-1. Recently, the stimulatory mechanistic effect of IGF-1 on testosterone production by sheep Leydig cells was reported [73]. In the present study, significant decreases in concentrations of T3 and T4 at post-puberty age (M4–M5) compared with pre-puberty (M1–M3) age may be attributable to the change of metabolic activity. Our results were consistent with those reported in cattle [74]. Although FSH did not show significant changes in the present study, it cannot be ascertained that the hypothalamus-pituitary axis was not affected, because we were solely dependent on measuring monthly changes in the basal FSH levels. However, measuring the FSH pulsatile secretion should also be considered, and that was not addressed in this experiment. Interestingly, decreased levels of LH at the 4th and 5th month post-puberty were found compared with pre-puberty age (M1–M3). These results were in agreement with the results obtained by [75]. However, these findings must be interpreted with caution because they may be owed to the utilization of LH for the synthesis of testosterone by Leydig cells at the attainment of the pubertal age. In addition, we measured the monthly basal LH concentrations (as FSH), while the LH pulsatile levels were not considered in the current study. In the present study, the concentrations of inhibin in the plasma during the first 5 months of age correlated positively with the levels of FSH and negatively with the levels of testosterone. Similar to the LH, inhibin concentrations were significantly higher during the first 3 months and then decreased at the 4th and 5th month. These findings were similar to those reported by Miyamoto et al. [71].

The oxidative stress markers in the current study revealed age-related changes. Even though these changes are minimal, the reported level of these markers was lowest at M1. In addition, GPX was significantly elevated with age advance, which matched the trend of plasma Se concentration until three months. Notably, in our study, MAD, SOD, and GPX were elevated from the third month, and at the fifth month, MDA, CAT, and SOD reduced, while GPX remained elevated. This may reflect the increased oxidative stress and lipid peroxidation after weaning toward puberty, depicting the importance of oxidative stress during the neonatal and peri-puberty stage. In the biological system, the balance between antioxidants, including but not limited to CAT, SOD, and GPX, and levels of oxidants (such as MDA) are important to scavenge the oxidative metabolites or to repair the consequent damage by oxidative stress in various tissues including hematopoietic and reproductive organs [76,77,78]. On the contrary, Cetin et al. [19] reported that oxidants and antioxidants do not change with regard to the dynamics of some reproductive hormones during the gestation period in black hairy goats. In addition, Celi et al. [79] stated that the oxidative stress marker responded differently, resulting in a lack of correlation with other parameters during the peripartum period in goats. Copious studies demonstrate changes in oxidative stress markers concerning various physiological states including pregnancy, lactation, and feeding regime in goats [3,76,80,81], rather than the changes concerning age in the growing period of kids. Various factors are incriminated in the fluctuation of oxidative stress markers. Trace minerals, particularly Cu and Zn, constitute a functional component to maintain SOD activity, whereas Se is closely related to GPX [82]. Unfortunately, most ruminant studies on oxidative stress are related to single insults, especially diseases and nutrition regimes, and physiological alterations in these parameters are still lacking [81]. Here, we have to say that the fluctuation in the levels of the reported measurements in the current study in association with oxidative stress markers could be ascribed to different stressors associated with changes in the metabolic states due to weaning, the active phase of development of body organs, reproductive changes associated with puberty, and environmental insults necessary for immunologic maturation. All these factors differentially affect the laboratory results; therefore, the correlation results between the measured variables were also variable.

Study limitations: The small sample size in this study should be considered; however, the population of goats in Japan is limited, since goats are frequently used for research and educational purposes rather than being a source of human food [5]. Our investigation was done only in male goats, which were principally kept in our lab for andrological studies, and therefore the sex difference of the studied parameters was not provided. The effect of environmental changes on the obtained measurements was not investigated. 

## 5. Conclusions

This study reported the values of basic hematology, plasma minerals, reproductive hormones, and oxidative and antioxidative markers in male goats from the first month toward the puberty age, which can support the laboratory interpretation of diseases and help in the evaluation of the efficiency of treatment, specifically in the terms of age in months or peri-pubertal changes.

## Figures and Tables

**Table 1:** Average monthly climatic data (February–June) during the study.

	Time of Sampling				
	M1	M2	M3	M4	M5
Temp (°C)	6.9	8.5	14.7	20.0	22.0
RH%	67	69	70	76	83
Wind-speed	4.6	4.0	5.3	5.6	5.4
THI (°C)	4.41	6.66	13.28	20.04	22.4

**Table 2:** Chemical composition of alfalfa hay cubes.

Ingredients	Concentration
Ash	2%
Crude protein	18%
Crude fiber	5.44%
Dm	91.5%
Ether extract	7.63%
Moisture	8.1%
Nitrogen-free extract	58.83%
Organic matter	88.64%
Calcium (Ca)	6.3 gm/kg Dm
Cobalt (Co)	0.3 mg/kg Dm
Chromium (Cr)	1.9 mg/kg Dm
Copper (Cu)	8.5 mg/kg Dm
Iron (Fe)	191.5 mg/kg Dm
Potassium (K)	4.9 gm/kg Dm
Magnesium (Mg)	2.1 gm/kg Dm
Manganese (Mn)	48.1 mg/kg Dm
Molybdenum (Mo)	0.5 mg/kg Dm
Sodium (Na)	2. 6 gm/kg Dm
Selenium (Se)	0.5 mg/kg Dm
Zinc (Zn)	37.0 mg/kg Dm

**Table 3:** Clinical parameters in healthy newborn kids during the first five months of life.

Parameter	Age (Month)				
	M1	M2	M3	M4	M5
Respiratory rate	71.0 ^a^ ± 7.0	64.0 ^b^ ± 4.0	55.0 ^b^ ± 5	51.0 ^b^ ± 4.0	57.0 ^b^ ± 4.0
Heart rate	150.0 ^a^ ± 10.0	119 ^ab^ ± 6.0	116.0 ^ab^ ± 10.0	96.0 ^b^ ± 9.0	87.0 ^b^ ± 9.0
B. temperature	39.5 ^a^ ± 0.3	38.7 ^a^ ± 0.7	39.0 ^a^ ± 0.2	39.4 ^a^ ± 0.4	39.6 ^a^ ± 0.4
Body weight	5.48 ^b^ ± 0.88	8.92 ^ab^ ± 1.25	9.28 ^ab^ ± 1.34	9.92 ^a^ ± 1.19	11.52 ^a^ ± 1.32

Different letters revealed significance between ages per month (*p* < 0.05).

**Table 4:** ICP-MS setting conditions.

Working Condition	Setting
Plasma gas flow	15.0 L/minute
Auxiliary gas flow	0.90 L/minute
Nebulizer gas flow	0.90 L/minute
Sample flow rate	0.55 mL/minute
HF-power	1.60 kW
Nebulizer type	Micro Mist
Sampling depth	9 mm
Integration time	100 msec
Scans	3 times
Scanning mode	Spectral analysis
Total analysis time	78 h
**Measured Isotopes**	
Trace elements	^63^Cu, ^66^Zn, ^78^Se,^56^Fe, ^52^Cr, ^59^Co, ^55^Mn,^95^Mo
Macroelements	^44^Ca, ^24^Mg

**Table 5:** Hematological profile during the first five months of life in male Shiba goats.

Variables	Age (Month)					*p* Value
	M1	M2	M3	M4	M5	
RBC (10^6^)	13.3 (12–16) ^a^	12.9 (8.56–14.96) ^a^	12.8 (6–16) ^a^	11.8 (7.7–13.9) ^a^	11.7 (7.6–17.3) ^a^	NS
Hb (gm/dL)	9.9 (7.6–12.5) ^a^	10.45 (8.9–11.7) ^a^	9.8 (9–13) ^a^	8.6 (6.6–12.4) ^a^	10 (8.2–11.3) ^a^	NS
HCT %	34.4 (25–48) ^a^	33.2 (30.5–42.4) ^a^	33 (30–38.8) ^a^	29 (22.2–38)^b^	31.4 (27–38) ^ab^	*
MCV(fl)	27.2 (21.4–37.2) ^a^	28.5 (22.5–37.2) ^a^	26.8 (19.6–55.2) ^a^	26.4 (20.5–31.6) ^a^	26.5 (19.1–39.2) ^a^	NS
MCH (pg)	7.6 (4.69–10.3) ^a^	7.9 (6.2–11.9) ^a^	8.6 (5.9–16.3) ^a^	7.8 (6.5–9.1) ^a^	8 (4.9–14.9) ^a^	NS
MCHC (fl)	29.2 (26.1–30.3) ^a^	28.1 (26.5–29.5) ^a^	28 (26.3–29.6) ^a^	29 (26–30.5) ^a^	29 (27.7–30.4) ^a^	NS
RDW%	20 (17.9–23.4) ^a^	21 (19.3–22.7) ^a^	19.9 (18–22.2) ^ab^	19.8 (18–23.5) ^bc^	19 (18.1–20.15) ^c^	**
PLT (10^4^)	16.7 (9.5–34.2) ^b^	25 (12.3–48.7) ^ab^	39.5 (14.4–75.5) ^a^	38 (13.4–82.1) ^a^	23.3 (15.7–55.4) ^ab^	*
PCT%	0.06 (0.03–0.13) ^c^	0.09 (0.04–42.4) ^bc^	0.14 (0.05–0.26) ^ab^	0.13 (0.05–0.3) ^ab^	0.08 (0.05–0.19) ^bc^	*
MPV (fl)	3.8 (3.3–4) ^a^	3.5 (2.7–3.9) ^ab^	3.4 (3.3–3.5) ^bc^	3.5 (3.4–3.7) ^ab^	3.4 (3.4–3.6) ^c^	**
PDW%	7.4 (6.5–8.7) ^a^	6.8 (5.9–7.6)^b^	7.3 (6.6–7.4) ^ab^	7 (6.2–8.1) ^a^	7.3 (6.5–7.6)^b^	*
WBC (10^2^)	154 (91–195) ^a^	186 (103–249) ^a^	166 (108–215) ^a^	167 (137–239) ^a^	184 (101–237) ^a^	NS
Neut %	29 (24–41) ^ab^	25.7 (6.52–33) ^b^	35 (7–45) ^ab^	36.7 (20–55) ^a^	32 (23–46) ^ab^	NS
Lymph %	57.4 (56–68) ^b^	70.6 (65–79) ^a^	67 (54–95) ^a^	62.6 (38–80) ^ab^	63.7 (48–74) ^ab^	*
Mono %	1.0 (1–12) ^a^	1.3 (0–6) ^a^	0.0 (0–5) ^ab^	0.0 (0–2) ^b^	0.0 (0–2) ^b^	**
Esino %	3.0 (2–7) ^a^	1.8 (0–3) ^bc^	1.0 (0–4) ^c^	2.0 (0–5) ^bc^	2.0 (2–6) ^ab^	**

Values are expressed as median and range (*n* = 16). Different letters revealed significance between ages per month (*p* < 0.05). NS, not significant, * *p* < 0.05, ** *p* < 0.01. Abbreviations: Hb, hemoglobin; RBC, red blood cell count; HCT, hematocrit; MCV, mean corpuscular volume; MCH, mean corpuscular hemoglobin; MCHC, mean corpuscular hemoglobin concentration; RDW, red blood cell distribution width; PLT, platelet count; PCT, plateletcrit; MPV, mean platelet volume; PDW, platelet distribution width; WBC, white blood cell count, Neut; neutrophils; Lymph, Lymphocytes; Mono, monocytes; Esino, esinophils.

## Data Availability

The data presented in this study are available on request.

## Supplementary Materials

The following supporting information can be downloaded at: https://www.mdpi.com/article/10.3390/ani12050645/s1. Table S1. Plasma minerals, oxidatve stress markers, and hormonal profile during the first-five months of life in male Shiba goats.
