# Supplementary material for: Monthly Dynamics of Plasma Elements, Hematology, Oxidative Stress Markers, and Hormonal Concentrations in Growing Male Shiba Goats (Capra hircus) Reared in Tokyo-Japan
Source: Animals (Basel). 2022 Mar 3;12(5):645. doi: 10.3390/ani12050645 (PMC8909858; doi:10.3390/ani12050645)
Supplement: Supplementary file 1 [file animals-12-00645-s001.zip › animals-1564685-supplementary.pdf]

Supplementary Table S1. plasma minerals, oxidatve stress markers, and hormonal profile during the first-five months of life in male Shiba goats

Variables	Age (month)					P value
	M1	M2	M3	M4	M5	
Plasma elements						
Cu (mg/L)	0.890 (0.51–1.365)^{a}	0.854 (0.741–1.357)^{a}	0.9525 (0.757–1.125)^{a}	0.707 (0.002–0.991)^{b}	0.701 (0.461–0.969)^{b}	0.0001***
Zn (mg/L)	0.645 (0.367–1.2)^{a}	0.607 (0.301–1.643)^{a}	0.569 (0.206–0.902)^{a}	0.569 (0.172–1.018)^{a}	0.474 (0.169–0.755)^{a}	0.185
Se (mg/L)	0.062 (0.029–0.097)^{a}	0.0645 (0.043–0.127)^{a}	0.0605 (0.031–0.179)^{a}	0.054 (0.027–0.325)^{a}	0.044 (0.023–0.154)^{a}	0.292
Fe (mg/L)	2.986 (1.75–5.83)^{a}	2.638 (2.103–4.492)^{ab}	2.18 (1.646–4.078)^{bc}	2.236 (1.305–3.648)^{c}	1.969 (1.215–3.139)^{c}	0.0001***
Cr (mg/L)	0.042 (0.013–0.059)^{b}	0.048 (0.027–0.111)^{ab}	0.0585 (0.039–0.107)^{a}	0.06 (0.015–0.127)^{a}	0.045 (0.029–0.147)a	0.045*
Ca (mg/L)	44.65 (29.12–68.31)^{a}	43.52 (36.03–57.04)^{a}	42.33 (32.24–56.52)^{ab}	43.57 (22.66–50.3)^{ab}	33.61 (22.22–52.58)^{b}	0.006**
Mg (mg/L)	23.3 (14.05–34.61)^{a}	23.28 (17.71–33.7)^{a}	25.3 (17.42–28.49)^{a}	23.51 (12.36–30.81)^{a}	15.59 (11.45–20.01)^{b}	0.0001***
Oxidative stress markers						
MAD (nmol/mL)	17.35 (15.4–23.6)^{a}	15.6 (13.1–19.4)^{a}	17.5 (13.3–24)^{a}	17.35 (13.3–23.1)^{a}	16.1 (13.8–21.6)^{a}	0.368
CAT (u/L)	278 (222–359)^{b}	351 (233–417)^{a}	339 (270–452)^{a}	304 (245–480)^{ab}	281 (252–355)^{ab}	0.043*
SOD (u/ml)	3.84 (3.13–4.11)^{b}	4.02 (3.2–5.27)^{ab}	4.28 (3.1–6.16)^{a}	4.13 (3.59–6.36)^{a}	3.75 (3.52–4.48)^{ab}	0.140
GPX (u/mL)	131.5 (116–147)^{c}	141.5 (121–164)^{bc}	142 (127–180)^{ab}	151.5 (138–189)^{a}	154 (133–179)^{a}	0.001**
Plasma hormones						
Cortisol (ng/mL)	23.34 (9.95–31.02)^{ab}	15.84 (7.514–42.24)^{b}	21.73 (6.361–55.37)^{ab}	21.44 (12.03–38.87)^{ab}	24.56 (10.98–49.23)^{a}	0.124
FSH (ng/mL)	0.874 (0.421–1.53)^{a}	0.442 (0.267–3.049)^{a}	0.993 (0.2401–3.014)^{a}	0.673 (0.1257–2.73)^{a}	0.439 (0.054–2.342)^{a}	0.489
LH (ng/mL)	0.548 (0.0357–1.862)^{a}	0.359 (0.1–1.292)^{ab}	0.259 (0.0778–1.973)^{ab}	0.169 (0.1–0.735)^{b}	0.198 (0.083–1.034)^{b}	0.039*
IGF1 (ng/mL)	141.9 (80–251.2)^{c}	132 (78–361.9)^{bc}	212.2 (130.9–321.2)^{a}	203.5 (72.67–311)^{ab}	140 (91.39–223.9)^{c}	0.007**
Inhibin (ng/mL)	10.61 (2.215–18.55)^{ab}	10.67 (2.629–20.95)^{a}	12.99 (7.288–19.95)^{a}	8.966 (2.876–17.52)^{a}	8.129 (6.286–13.73)^{b}	0.011*
Testosterone (ng/mL)	159 (20.5–639.2)^{b}	426 (80.66–995.2)^{a}	280.1 (38.33–816)^{ab}	398.7 (94.98–900)^{ab}	431.5 (144.4–700)^{a}	0.046*
T3 (nmol/L)	17.68 (8.52–23.55)^{a}	12.57 (11.45–14.8)^{bc}	15.64 (7.83–18.9)^{ab}	10.02 (5.37–17.68)^{cd}	9.72 (5.18–14)^{d}	0.0001***
T4 (nmol/L)	11.3 (6.1–18.7)^{a}	9.15 (8–11.5)^{bc}	10.95 (5.6–12.9)^{ab}	7.05 (3.9–12.7)^{cd}	7 (3.7–9.8)^{d}	0.0001***

Values are expressed as median and range (n = 16). * $P < 0.05$, ** $P < 0.01$, ***$P < 0.001$. Abbreviations: Cu, copper; Zn, zinc; Fe, iron; Se, selenium; Cr, chromium; Ca, calcium; Mg, magnesium; MDA, malondialdehyde; CAT, catalase; SOD, superoxide dismutase; GPX, glutathione peroxidase; IGF1, insulin-like growth factor 1; T3, triiodothyronine; T4, tetraiodothyronine; FSH, follicular stimulating hormones; LH, leutinizing hormone.
